# Diagnostic accuracy of magnetic resonance elastography and point-shear wave elastography for significant hepatic fibrosis screening: Systematic review and meta-analysis

**DOI:** 10.1371/journal.pone.0271572

**Published:** 2023-02-02

**Authors:** João Paulo L. Schambeck, Gabriele C. Forte, Luana M. Gonçalves, Guilherme Stuker, João Bruno F. Kotlinski, Giacomo Tramontin, Stephan Altmayer, Guilherme Watte, Bruno Hochhegger

**Affiliations:** 1 Post-Graduate Program in Medicine and Health Science, Pontifícia Universidade Católica do Rio Grande do Sul, Porto Alegre, Rio Grande do Sul, Brazil; 2 Departament of Radiology, Hospital São Lucas/Pontifícia Universidade Católica do Rio Grande do Sul, Porto Alegre, Rio Grande do Sul, Brazil; 3 Faculty of Medicine, Pontifícia Universidade Católica do Rio Grande do Sul, Porto Alegre, Rio Grande do Sul, Brazil; 4 Department of Radiology, Medical Imaging Research Lab, LABIMED, Porto Alegre, Rio Grande do Sul, Brazil; 5 Department of Diagnostic Methods, Federal University of Health Sciences of Porto Alegre, Porto Alegre, Rio Grande do Sul, Brazil; Kaohsiung Medical University, TAIWAN

## Abstract

The hepatic diseases are extremely common in clinical practice. The correct classification of liver fibrosis is extremely important, as it influences therapy and predicts disease outcomes. The purpose of this study is to compare the diagnostic performance of point-shear wave elastography (pSWE) and magnetic resonance elastography (MRE) in the hepatic fibrosis diagnostic. A meta-analysis was carried out based on articles published until October 2020. The articles are available at following databases: MEDLINE, EMBASE, Cochrane Central Register of Controlled Trials, Scientific Electronic Library Online, LILACS, Scopus, and CINAHL. Diagnostic performances were analyzed per METAVIR F2, using 3.5kPa as target fibrosis. Assessment of the methodological quality of the incorporated papers by the QUADAS-2 tool for pSWE and MRE. A total 2,153 studies articles were evaluated and 44 studies, comprising 6,081 patients with individual data, were included in the meta-analysis: 28 studies for pSWE and 16 studies for MRE. The pooled sensitivity and specificity were 0.86 (95%CI 0.80–0.90) and 0.88 (95%CI 0.85–0.91), respectively, for pSWE, compared with 0.94 (95%CI 0.89–0.97) and 0.95 (95%CI 0.89–0.98) respectively, for MRE. The pooled SROC curve for pSWE shows in the area under the curve (AUC) of 0.93 (95%CI 0.90–0.95), whereas the AUC for MRE was 0.98 (95%CI 0.96–0.99). The diagnostic odds ratio for pSWE and MRE were 41 (95%CI 24–72) and 293 (95%CI 86–1000), respectively. There was statistically significant heterogeneity for pSWE sensitivity (I² = 85.26, P<0.001) and specificity (I² = 89.46, P<0.001). The heterogeneity for MRE also was significant for sensitivity (I² = 73.28, P<0.001) and specificity (I² = 87.24, P<0.001). Therefore, both pSWE and MRE are suitable modalities for assessing liver fibrosis. In addition, MRE is a more accurate imaging technique than pSWE and can be used as alternative to invasive biopsy.

## Introduction

The hepatic diseases are extremely common in clinical practice [1]. Constant cell damage can lead to progressive fibrosis and, consequently, to the final stage, cirrhosis [2]. The right staging is extremely important given that the amount of fibrosis influences the therapy and predicts the diseases outcomes [3, 4]. Even in the final stage, the patient may remain “compensated” for months or years. However, after cirrhosis is established, it is estimated that the annual mortality rates can reach 57% [5].

For the impairment grading of liver parenchyma and diagnosis of fibrosis, liver biopsy is still considered the reference standard. However, it is an invasive technique that requires some considerations. Hospitalization for several hours is needed [5]. Although the fibrosis involvement tends to be diffuse, it does not have a uniform distribution in the hepatic parenchyma and we often see some areas more affected by fibrosis than others [6–9]. Besides, intra- and inter observer variability is another limitation which may lead to misdiagnosis and incorrect staging [10, 11]. In light of this, a liver biopsy may have uncertain accuracy, feasibility, and reliability [12]. Consequently, non-invasive techniques are tempting for avoid iatrogenic complications, being a safer approach for the follow-up monitoring [12].

Among the alternatives, we emphasize the elastography techniques, which are based on the measurement of mechanical properties of the interested tissues [4, 13, 14]. A decrease in elasticity may represent more advanced fibrosis staging. Point-shear wave elastography (pSWE) is an ultrasound-based evaluation with easy access, quick attainment, and low cost. It is able to measure shear wave velocity estimating the tissue stiffness, as well a simultaneous evaluation of the inner structures of the liver and surrounding [4, 15]. The required equipment is becoming progressively more compact, which allow inpatient and outpatient evaluation. However, this method has some limitations, such as being operator dependent, which may lead to inter and intra-observer variance, and the evaluation is considerably impaired in patients with ascites and obesity [16].

Magnetic resonance elastography (MRE) is another attractive approach as non-invasive assessment [4, 13]. Beyond the stiffness measurement using complex algorithms, it offers the possibility of morphological study of the entire liver and upper abdomen. MRE is becoming more assessable, although the cost is relatively higher than the pSWE study.

The aim of this meta-analysis was to compare the diagnostic performance of pSWE and MRE for the diagnostic of hepatic fibrosis.

## Material and methods

This systematic review and meta-analysis were performed in accordance with the PRISMA (Preferred Reporting Items for Systematic Reviews and Meta-Analyses) statement guidelines [17]. A protocol was designed a priori and registered at PROSPERO: International prospective register of systematic reviews (*PROSPERO 2020 CRD42020162774)*. PIRO (P = adult patients; I = MRE and pSWE; R = Liver biopsy (METAVIR score); O = hepatic fibrosis).

### Search strategy

MEDLINE (via PUBMED), EMBASE, Cochrane Central Register of Controlled Trials (CENTRAL, The Cochrane Library), Scientific Electronic Library Online (SciELO), LILACS, Scopus, and CINAHL database were searched through October 2020. Reference list of identified studies and reviews were also hand-searched. The search strategy included the descriptors (MeSH terms and other entry terms) related to pSWE, MRE, METAVIR, and hepatic fibrosis (S1 File).

#### Eligibility criteria

Full papers without language restrictions that evaluated pSWE or MRE in the diagnosis of liver fibrosis (stage 2), using liver biopsy as the reference standard and classified according to METAVIR score were included.

The following exclusion criteria were used: (a) duplicated publications or studies additional to those already included; (b) biopsy proven which uses other than METAVIR score; (c) study not published; (d) case reports, letters to the editor, reviews, abstracts and meta-analysis; (e) study not available; (f) study with other outcomes than hepatic fibrosis (stage 2 or higher); (g) study with insufficient data for 2x2 table; (h) studies that evaluated exclusively nonalcoholic fatty liver disease (NAFLD).

### Study selection

Two investigators (G.S. and G.T.) independently reviewed the titles and abstracts of each article identified in the literature search. All articles that clearly did not meet the inclusion criteria were excluded. The selected articles were retrieved for full-text analysis and eligible articles were identified. In case of disagreement, the articles were reviewed aiming at a consensus position, and if no consensus could be achieved, a third investigator resolved discrepancies (G.C.F.).

#### Data extraction

Extraction of data from each study included in this review was also conducted independently by two investigators (J.B.F.K and L.M.G.), using a standardized instrument. The following data were extracted: country of study’s origin, year of publication, study design, patient number, patient age, sex and body mass index, technical failures in undertaking liver elastography, histological score used, true positive, true negative, false positive, and false negative pSWE and MRE results.

#### Methodological quality assessment

Two reviewers independently performed the quality assessment of the RCTs according to Quality Assessment of Diagnostic Accuracy Studies (QUADAS)-2 tool [18]. The patient selection, index test, reference standard, and flow and timing domains were evaluated. This tool classifies studies as low-risk (if most of the information is classified as having a low risk of bias), uncertain-risk (if reporting is insufficient to allow assessment), or high-risk (if the proportion of high-risk information is sufficient to affect interpretation of study results). A third reviewer (J.P.L.S.) resolved discrepancies between the two reviewers.

### Statistical analysis

The pooled sensitivities, specificities, and 95% confidence intervals (CIs) were calculated by using random-effect analysis. The pooled positive likelihood ratio (PLR), negative likelihood ratio (NLR), and diagnostic odds ratios (DORs) were also obtained. Summary receiver operating characteristic curves were constructed, and the areas under the curve were obtained. To assume an approximate normal distribution, we used the distribution of logit-transformed sensitivity and specificity and the natural logarithm of DOR. Heterogeneity for pooled sensitivities and specificities was calculated in terms of I^2^. The threshold effect was quantified using Spearman’s correlation coefficient between logit sensitivity and logit specificity and a coefficient (ρ) ≥ -0.6 was considered significant. If the threshold effect was not significant, further subgroup analysis stratifying for study characteristics was planned to identify potential sources of heterogeneity for each imaging modality if at least three studies met the subgroup characteristics. A likelihood ratio test was used to compare the regression models of subgroups. A continuity correction of 1 was used when calculating the logit transformed sensitivity and specificity. The Deeks funnel plot was used to display possible publication bias. Interstudy heterogeneity was also evaluated by using Galbraith plots. All analyses were performed by using Stata, version 12.0 (Stata, College Station, Tex).

## Results

**Fig 1:**
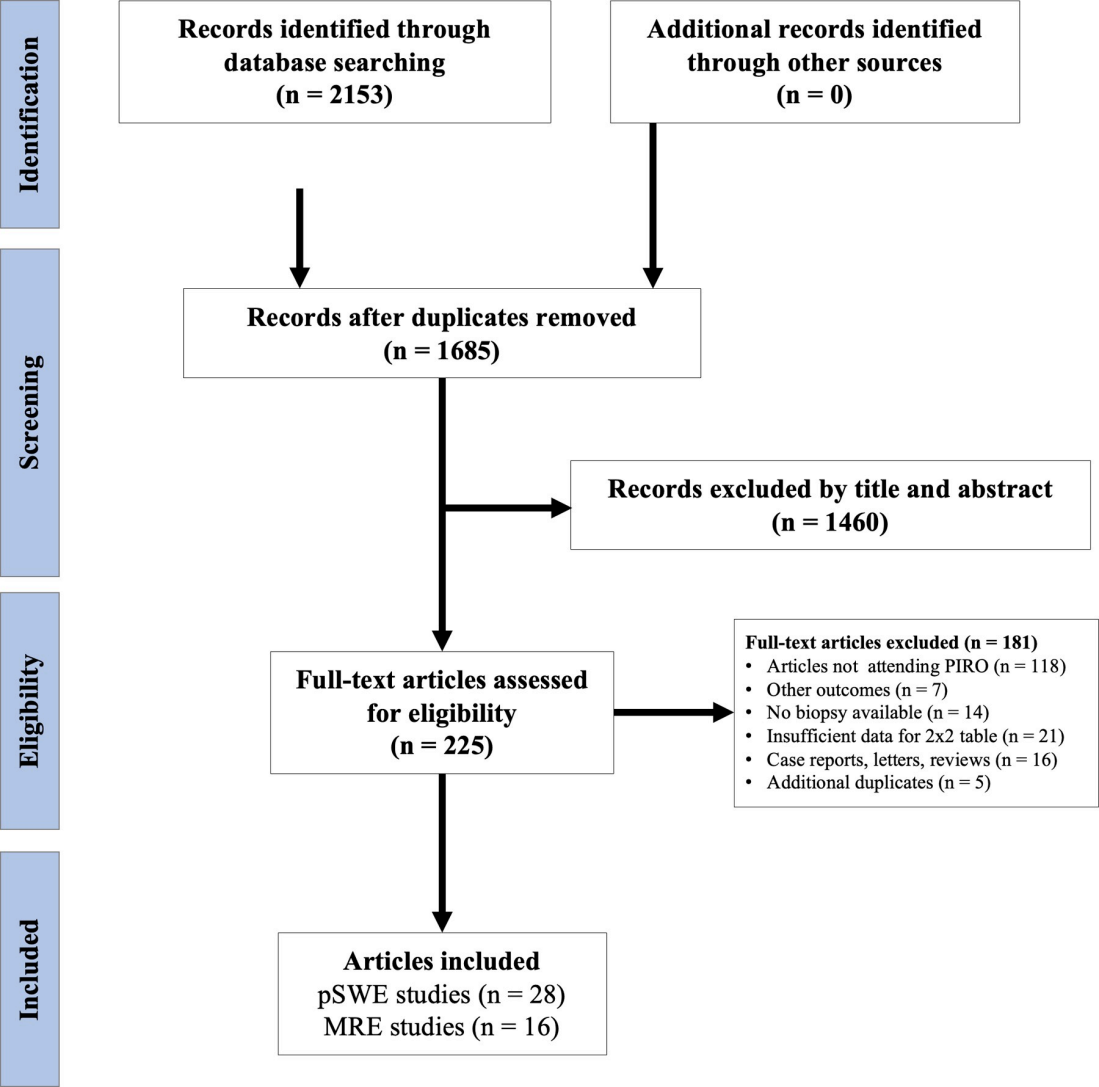
Study selection for meta-analysis. Point-shear wave elastography (pSWE). MRI = magnetic resonance imaging.

The initial search returned 2,153 studies, from which 468 were duplicate. We screened the remaining 1,685 titles and abstracts of which 1,460 were excluded. Of 225 articles full-text articles assessed for eligibility, we excluded 180 studies. Finally, 44 studies, comprising 6,081 patients with individual data, were included in the meta-analysis: 28 studies for pSWE and 16 studies for MRE (Fig 1).

Table 1 contains the main features of the pSWE studies included in this systematic review and meta-analysis. Most of the studies were conducted in European countries, followed by Asian countries. There were a total of three in Italy [19–21], four in Romania [22–25], two in France [26–28], one in Spain [29], one in Indonesia [30], and two German [31, 32], two in Brazil [33, 34], one in United State [35], seven in China [36–42], three in Japan [43–45], one in Egypt [46], and one in South Korea [47]. Many of the studies were prospective in design and performed in a single center. The mean age of the 4,465 patients was 52.8 years [SD 2.8], with a predominance of men (n = 2,331, 52.2%), and a mean body mass index was 24.9 kg/m² (SD 1.1). A total of 14 studies included patients with viral etiology only [20, 23, 25, 30, 32–34, 36–39, 41, 43, 46]. The other 14 studies were performed mostly in a mixed set of patients, including viral etiologies, autoimmune liver diseases (primary biliary cirrhosis, autoimmune hepatitis, among others), alcoholic cirrhosis, and small subset of patients with NALFD [19, 21, 22, 24, 27–29, 31, 35, 40, 42, 44, 45, 47].

**Table 1:** General characteristics of the pSWE selected articles.

Author, year	Country	Study design	Center	Sample size	Mean age (y)	Male sex	BMI (kg/m²)
**Dhyani, 2018**	USA	Prospective	Single	20	54	12	ND
**Karlas, 2011**	Germany	Prospective	Single	97	42.7	68	24.0
**Nishikawa, 2014**	Japan	Prospective	Single	108	59.5	56	22.5
**Liu, 2015**	China	Prospective	Single	108	40.8	81	21.9
**Liu, 2017**	China	Retrospective	Single	174	36.8	107	ND
**Liu, 2016**	China	Prospective	Single	187	34.9	111	ND
**Lin, 2016**	Taiwan	Prospective	Single	60	51.8	40	26.7
**Colombo, 2012**	Italy	Prospective	Single	54	55	38	25.8
**Tomita, 2013**	Japan	Prospective	Single	22	6.3	13	ND
**Tai, 2015**	Taiwan	Prospective	Single	204	52.9	48	ND
**Gani, 2017**	Indonesia	Prospective	Single	43	47.3	31	ND
**Rust, 2009**	Germany	Prospective	Single	86	48	46	26
**Elhosary, 2016**	Egypt	Prospective	Single	190	53.3	142	ND
**Crespo, 2012**	Spain	Prospective	Single	146	54	90	25.5
**Chung, 2013**	South Korea	Prospective	Single	74	47.3	35	ND
**Chen, 2015**	China/Taiwan	Prospective	Single	137	54	63	24.1
**Chen, 2012**	China/Taiwan	Prospective	Single	142	51.6	59	24.6
**Cassinotto, 2014**	France	Prospective	Multiple	349	54.8	188	27.4
**Cassinotto, 2013**	France	Prospective	Single	321	54.4	192	27
**Takahashi, 2010**	Japan	Prospective	Single	55	59.9	30	23.5
**Sporea, 2010**	Romania	Prospective	Single	114	46.9	53	ND
**Sporea, 2011**	Romania	Prospective	Multiple	197	50	78	ND
**Sporea, 2012**	Romania	Retrospective	Multiple	914	55.7	423	24.7
**Sporea, 2011**	Romania	Prospective	Single	233	48	90	ND
**Silva, 2014**	Brazil	Prospective	Single	51	53.8	18	25.1
**Rizzo, 2011**	Italy	Prospective	Single	139	55	83	26
**Ragazzo, 2017**	Brazil	Prospective	Single	107	49.1	53	24.9
**Piscaglia, 2011**	Italy	Prospective	Single	133	58	83	ND

BMI = body mass index; US = ultrasound; ND = not described.

The characteristics of the MRE studies were summarized in Table 2. The study centers were located in Netherlands (n = 1) [48], in Belgium (n = 1) [49], in United State (n = 4) [50–52], in China (n = 2) [53, 54], in Taiwan (n = 2) [55, 56], in Singapore (n = 2) [57, 58], in South Korea (n = 2) [59, 60], and in Japan (n = 2) [61, 62]. Eight studies (50%) were prospective and fifteen were performed in single center. Twelve studies (75%) were performed with MRE 1.5 Tesla. Taken together, the studies reported data from 1,616 subjects. The mean age was 52.8 years (SD 7.6), with majority men (n = 1,000, 61.8%). The mean body mass index was 24.5 kg/m² (SD 1.5). There were 6 studies including only patients with chronic viral liver disease [48, 53–55, 57, 58], while the other 10 studies had a more diverse patient population including several etiologies of chronic liver disease in the same study [13, 49–52, 56, 59–62].

**Table 2:** General characteristics of the MR elastography selected articles.

Author, year	Country	Study design	Center	Sample size	Mean age (y)	Male sex	BMI (kg/m²)	Magnetic field (T)
**Kim, 2011**	South Korea	Prospective	Single	55	58.3	46	22.3	1.5
**Huwart, 2007**	Belgium	Prospective	Single	88	54	37	25	1.5
**Ye, 2012**	South Korea	Retrospective	Single	173	57.2	129	22.7	1.5
**Hennedige, 2017**	Singapore	Retrospective	Single	63	50.1	44	24.9	1.5
**Ichikawa, 2015**	Japan	Retrospective	Single	182	66.4	127	ND	3.0
**Shi, 2014**	China	Prospective	Single	113	42	48	21.7	3.0
**Toguchi, 2017**	Japan	Retrospective	Single	51	59.9	ND	ND	1.5
**Venkatesh, 2013**	Singapore	Prospective	Multiple	63	50	44	24.8	1.5
**Venkatesh, 2014**	USA	Retrospective	Single	62	54.6	31	ND	1.5
**Wu, 2017**	Taiwan	Retrospective	Single	104	60.6	87	24.5	1.5
**Bohte, 2014**	Netherlands	Prospective	Single	85	45	55	25.5	3.0
**Besa, 2018**	USA	Retrospective	Single	83	58.4	59	25.7	1.5
**Batheja, 2015**	USA	Prospective	Single	54	38.5	0	30	1.5
**Wu, 2015**	Taiwan	Retrospective	Single	185	53.2	135	24	1.5
**Wang, 2011**	USA	Prospective	Single	76	55	50	ND	1.5
**Shi, 2016**	China	Prospective	Single	179	42.9	108	23	3.0

BMI = body mass index; MR = magnetic resonance; T = Tesla; ND = not described.

### Quality appraisal

**Fig 2:**
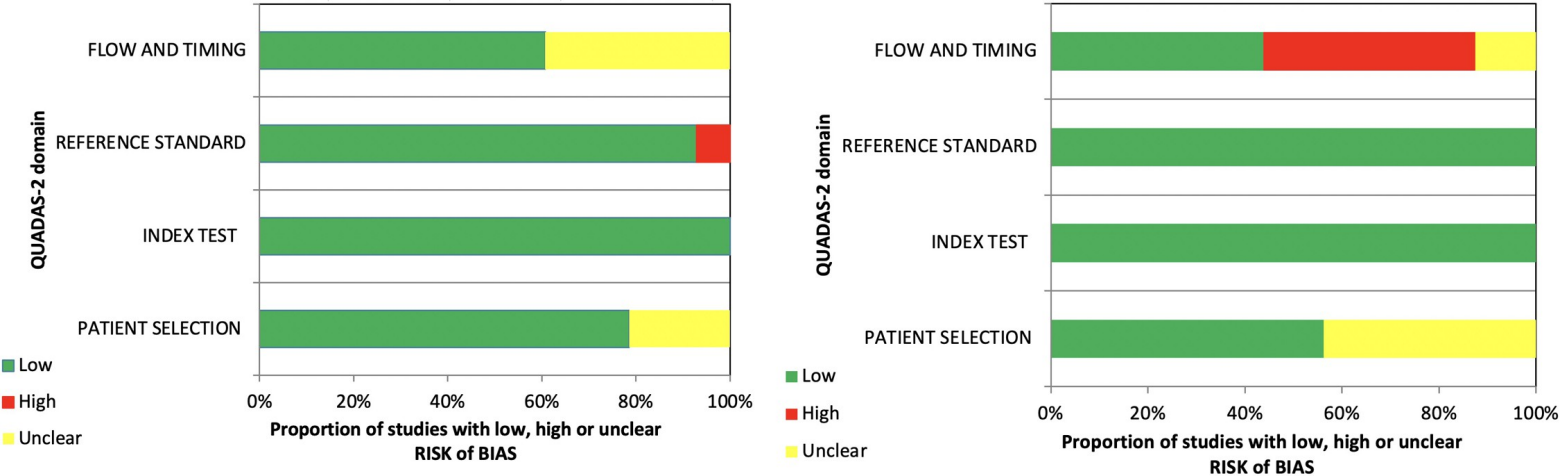
(A) Proportion pSWE and (B) MR elastography studies with low, high, and uncertain risk of bias according to the domains of the QUADAS-2 quality tool.

Assessment of the methodological quality of the incorporated papers by the QUADAS-2 tool for pSWE and MR elastography is depicted in Fig 2. In the “patient selection” domain, 31 studies were at relatively low risk of bias and 13 unclear. In “index test” domain, all studies were at low risk of bias. In “reference standard”, 42 studies were regarded as low risk and two were unclear. In terms of “flow and timing, 24 studies were scored with low risk of bias, seven, high risk, and 13 unclear.

### Diagnostic accuracy of hepatic fibrosis

**Fig 3:**
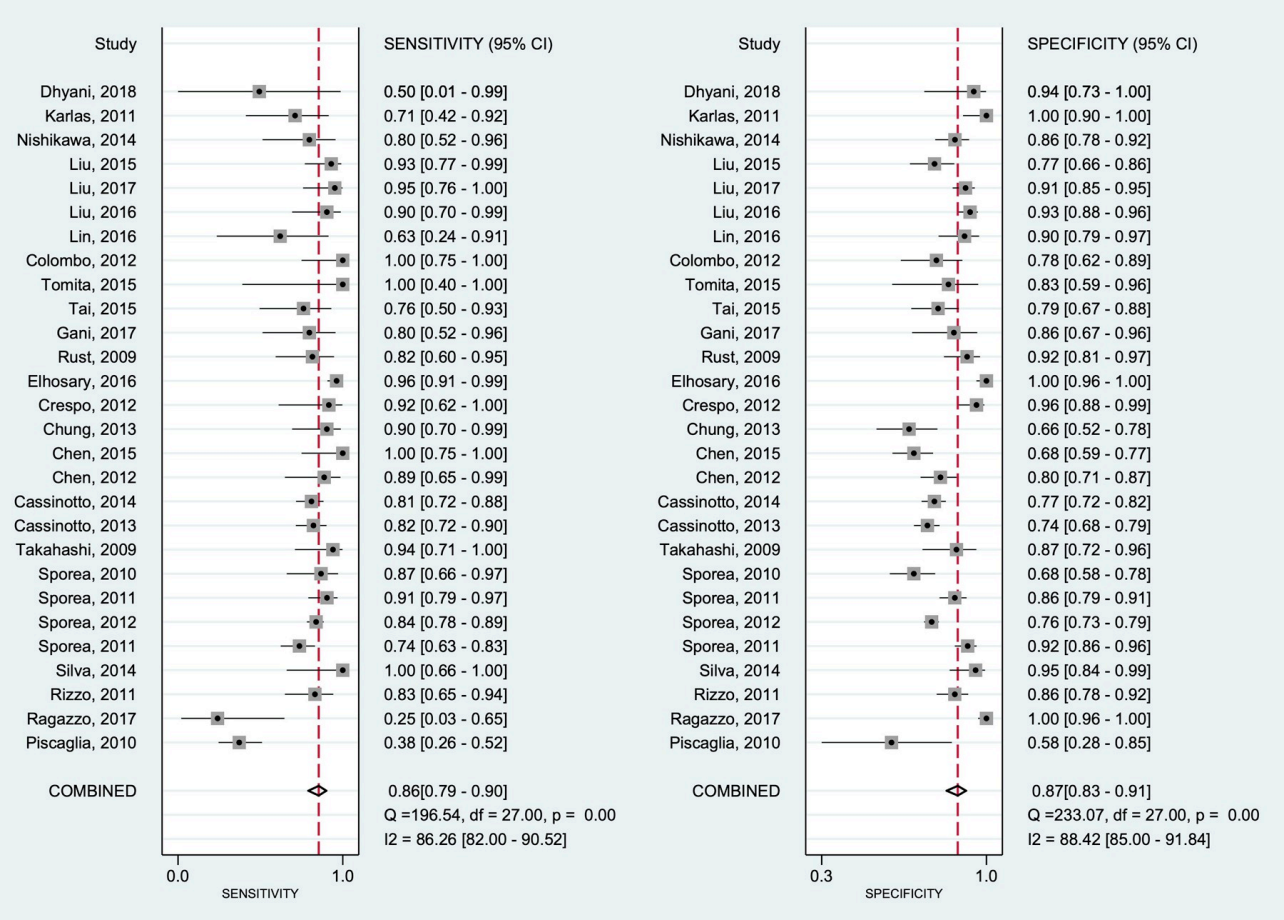
Forest plot of estimates of sensitivity and specificity of pSWE for diagnosis of hepatic fibrosis. The 95% confidence intervals (CI) are shown around point estimates and the pooled result. Plots show (A) sensitivity and (B) specificity of pSWE.

**Fig 4:**
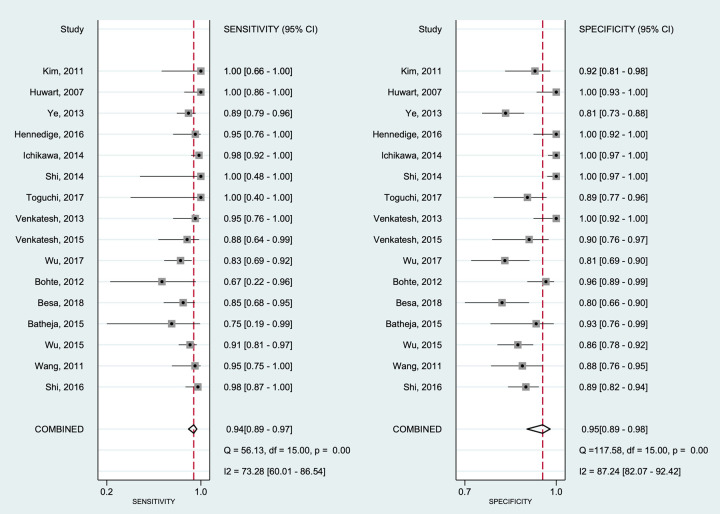
Forest plot of estimates of sensitivity and specificity of MR elastography for diagnosis of hepatic fibrosis. The 95% confidence intervals (CI) are shown around point estimates and the pooled result. Plots show (A) sensitivity and (B) specificity of MR elastography.

**Fig 5:**
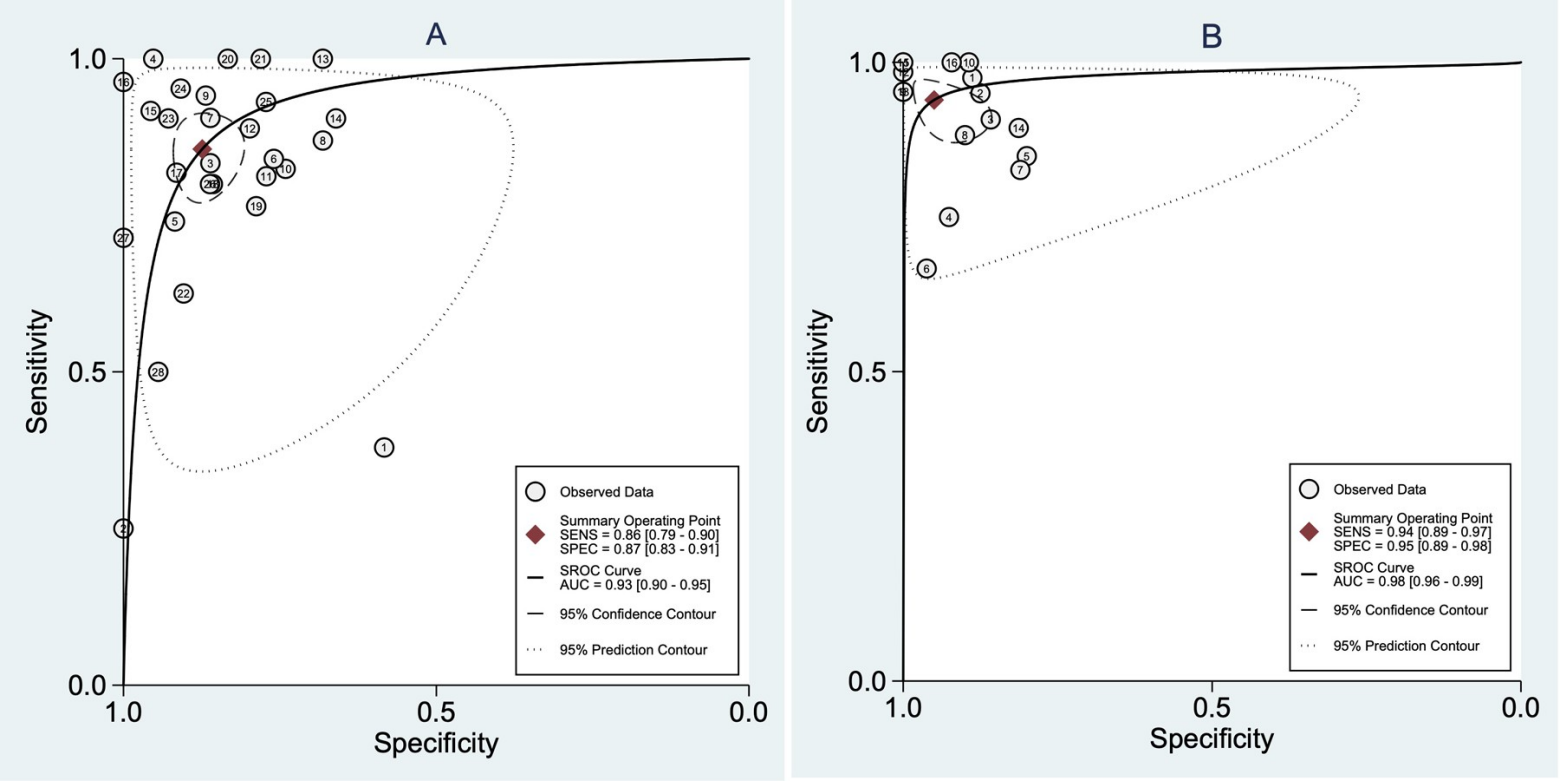
(A) Summarized receiver operating characteristic (SROC) curves for pSWE and (B) MRE for the diagnosis of hepatic fibrosis.

Diagnostic performances were analyzed per fibrosis (METAVIR F2) using 3,5 kPa as target fibrosis in all studies included. Forest plots for the sensitivities and specificities with theirs corresponding 95% confidence intervals (CI) of pSWE and MRE are shown in Figs 3 and 4, respectively. The pooled sensitivity and specificity were 0.86 (95%CI 0.79–0.90) and 0.87 (95%CI 0.83–0.91), respectively, for pSWE, compared with 0.94 (95%CI 0.89–0.97) and 0.95 (95%CI 0.89–0.98) respectively, for MRE. The pooled SROC curve for pSWE (Fig 5A) shows in the area under the curve (AUC) of 0.93 (95%CI 0.90–0.95), whereas the AUC for MRE was 0.98 (95%CI 0.96–0.99) (Fig 5B).

**Fig 6:**
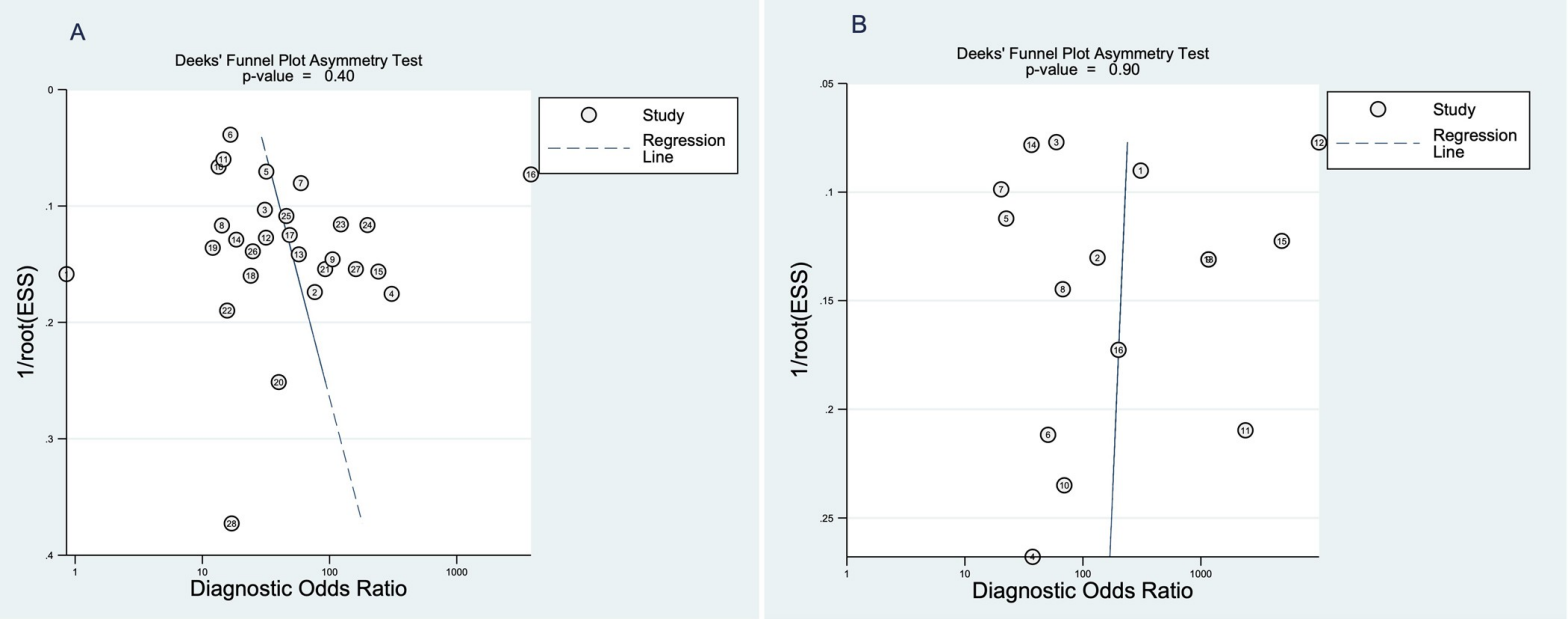
(A) Funnel plot for assessment of potential publication bias in the pSWE studies. (B) Funnel plot for the MRE studies.

The diagnostic odds ratio for pSWE and MRE were 41 (95%CI 24–72) and 293 (95%CI 86–1000), respectively. The Deeks’ funnel plot regression revealed no statistical evidence of asymmetry for pSWE (p = 0.40) and MRE (p = 0.90) (Fig 6), which suggests no asymmetry and major publication bias.

### Heterogeneity analysis and subgroup analysis

There was statistically significant heterogeneity for pSWE sensitivity (I² = 0.86, P<0.001) and specificity (I² = 0.88, P<0.001). The heterogeneity for MRE also was significant for sensitivity (I² = 0.73, P<0.001) and specificity (I² = 0.87, P<0.001). The threshold effect was neither significant for pSWE (ρ = -0.14), nor for MRE (ρ = 0.34). Therefore, subgroup analyses were conducted for both pSWE and MRE to investigate potential factors contributing to the heterogeneity.

The subgroup analysis for the pSWE studies (Table 3) revealed that the number of centers (multicentric vs. single) was a significant contributor to heterogeneity, with multicentric studies presenting lower sensitivity and specificity (p = 0.03). However, etiology of cirrhosis (p = 0.09) and the country where the study was performed (p = 0.07) also showed a trend towards different diagnostic performance between groups. Analysis by design (prospective vs. retrospective) was attempted, but only two studies were included in the retrospective group therefore the comparison was not conducted.

**Table 3:** Subgroup analyses of the diagnostic performance of pSWE for the evaluation of liver fibrosis (28 Studies).

Characteristics	No. of studies	Sensitivity (95% CI)	Specificity (95% CI)	p-value
Year publication				0.25
Before 2015	17	0.84 (0.77–0.89)	0.83 (0.77–0.88)	
≥ 2015	11	0.88 (0.73–0.95)	0.91 (0.83–0.96)	
Region of study				0.07
Non-Asia	15	0.79 (0.68–0.87)	0.89 (0.80–0.94)	
Asia	13	0.90 (0.85–0.93)	0.86 (0.79–0.91)	
Number of centers				0.03
Single center	25	0.86 (0.79–0.91)	0.88 (0.83–0.92)	
Multicenter	3	0.83 (0.72–0.91)	0.83 (0.76–0.89)	
Sample size				0.57
≥100	19	0.84 (0.77–0.90)	0.86 (0.79–0.91)	
<100	9	0.87 (0.77–0.93)	0.89 (0.81–0.94)	
Etiology of cirrhosis				0.09
Viral	14	0.86 (0.79–0.91)	0.89 (0.83–0.93)	
Mixed	14	0.83 (0.72–0.91)	0.83 (0.76–0.89)	

95%CI = 95% confidence interval; pSWE = point-shear wave elastography.

The subgroup analysis for MRE revealed that the study design significantly contributed to heterogeneity, as the studies with prospective design presented significantly higher sensitivity and specificity than the retrospective studies included (Table 4). Analysis regarding number of centers was not possible due to limited number of multicentric studies found in the literature.

**Table 4:** Subgroup analyses of the diagnostic performance of MRE for the evaluation of liver fibrosis (16 studies).

Characteristics	No. of studies	Sensitivity (95% CI)	Specificity (95% CI)	p-value
Year publication				0.09
Before 2015	8	0.96 (0.90–0.98)	0.98 (0.89–0.99)	
≥ 2015	8	0.91 (0.84–0.94)	0.88 (0.83–0.91)	
Design				0.001
Prospective	8	0.95 (0.88–0.98)	0.96 (0.90–0.99)	
Retrospective	8	0.92 (0.85–0.95)	0.92 (0.81–0.97)	
Region of study				0.57
Non-Asia	6	0.90 (0.82–0.95)	0.92 (0.84–0.96)	
Asia	10	0.94 (0.90–0.97)	0.95 (0.87–0.98)	
Sample size				0.70
≥100	6	0.95 (0.85–0.98)	0.95 (0.79–0.99)	
<100	10	0.92 (0.86–0.96)	0.94 (0.89–0.97)	
Etiology of cirrhosis				0.42
Viral	6	0.93 (0.87–0.97)	0.98 (0.85–0.99)	
Mixed	10	0.93 (0.86–0.97)	0.92 (0.84–0.96)	

95%CI = 95% confidence interval.

## Discussion

In the present meta-analysis, it was evaluated the diagnostic performance of ultrasound elastography, evaluated by pSWE and magnetic resonance elastography in the staging 2 of liver fibrosis, as reported in 44 studies (28 for pSWE and 16 for MRE). The use of METAVIR F2 (set by 3,5 kPa) as a cut-off value for pathologic findings dues to its importance in clinical practice: the begin of clinical treatment to reduce the progression of liver fibrosis. Both MRE and pSWE proved to be an important tool for early diagnosis of liver fibroses, reducing the role of biopsies by encompassing a greater part of liver parenchyma besides being a non-invasive diagnosis method, especially MRE according to our findings in this meta-analysis.

Our results showed that pSWE and MRE could be used to diagnose liver fibrosis. Both imaging methods provide excellent diagnostic accuracy for staging 2 liver fibrosis, with AUROC of 0.93 and 0.98 for pSWE and MRE, respectively. However, the sensitivity and specificity of MRE shows superior results compared to pSWE for the diagnosis of stage two of liver fibrosis. pSWE and MRE showed probability of 86% and 94%, respectively, correctly to diagnose liver fibrosis following a “positive” measurement.

Previous meta-analysis demonstrated inferior sensitivity and specificity compared to the present study, for both point-shear wave elastography and resonance elastography. Tsochatzis et al. [2] demonstrated accuracy of transient elastography for diagnose the severity of fibrosis in chronic liver disease. The summary sensitivity and specificity detected in stage F2 (31 studies) was 0.79 and 0.78, respectively. Su et al. [63] when assessing the accuracy of MRE for stage F2 liver fibrosis, showed results of sensitivity and specificity, respectively, 0.87 and 0.92. Guo et al. [6] show sensitivity 0.76 for pSWE and 0.87 for MRE, and significance was found in AUROC between pSWE (0.85) and MRE (0.97) for the diagnosis of stage 2 liver fibrosis.

Although in the study by Guo et al. [6] considerable heterogeneities were not observed in the MRE and pSWE studies, our study revealed significant heterogeneity in both imaging modalities for the evaluation of significant liver fibrosis. Tsochatzis et al. [2] showed results similar to the present meta-analysis finding statistically significant heterogeneity for stage 2 (I² = 67%, p<0.001), but not for the others. In our study, heterogeneity was not fully explained by threshold effect and further sub analysis was conducted. Three factors were shown to be related to heterogeneity in pSWE studies (number of centers, etiology, country of origin), although only the number of centers was statistically significant with multicentric presenting lower sensitivity and specificity than single center studies. For MRE, only the design of the studies were found to be in part contributing to the heterogeneity, with prospective studies demonstrating higher sensitivity and specificity compared to the retrospective group. Nonetheless, the summarized diagnostic performances of both modalities should be interpreted with caution due to high heterogeneity.

Although liver biopsy yet is the reference standard for evaluating and classifying stage of liver fibrosis, it has several limitations. It is invasive method and can cause minor complications including temporary pain until major complications, such as bleeding, hemothorax and even death [64, 65]. Accurate staging of liver fibrosis is very important, since hepatic fibrosis has a potential for reversal when in initial stages [66]. Therefore, the presence of significant fibrosis (F2) is already considered an important finding of progressive disease and needs special attention [67].

We adopted a systematic search and analysis strategy to assess the accuracy of pSWE and MRE for diagnose of significant liver fibrosis. However, there are still limitations in our meta-analysis. First, we have only included full-text analysis with histopathological score METAVIR. Second, we have not included patients with NAFLD to control for some of the hepatic inflammation which could have contributed to the heterogeneity of the studies, but it may limit some of the representativeness of our results. Third, there was significant heterogeneity in the meta-analysis of both modalities that were not fully accounted for the threshold effect. Our analysis was limited because there is not studies assessing joint pSWE and MRE in the same population. There is a single study that evaluated MRE and ultrasound by elastography, but it used the transient elastography instead of pSWE. Despite the heterogeneity and limitations found in this study, the meta-analysis results reported non-invasive clinical practice for the diagnosis of liver fibrosis. Furthermore, our study included 44 studies with a large sample size and most prospective design studies.

In conclusion, our meta-analysis shows pSWE and MRE provide excellent diagnostic accuracy for significant liver fibrosis. These methods, especially the MRE, can be used as an alternative to invasive biopsy. We suggest further studies with an adequate design and sample size comparing different elastography techniques.

## Supporting information

S1 File Search strategy. (DOCX)

S2 File PRISMA checklist. (PDF)
