# Supplementary material for: Diagnostic accuracy of magnetic resonance elastography and point-shear wave elastography for significant hepatic fibrosis screening: Systematic review and meta-analysis
Source: PLoS One. 2023 Feb 2;18(2):e0271572. doi: 10.1371/journal.pone.0271572 (PMC9894488; doi:10.1371/journal.pone.0271572)
Supplement: S1 File — (DOCX) [file pone.0271572.s001.docx]

**Supporting information**

**Supporting file 1. Search strategy**

(((Magnetic Resonance Elastography) OR Elastographies, Magnetic Resonance) OR Elastography, Magnetic Resonance) OR Magnetic Resonance Elastographies) OR Resonance Elastographies, Magnetic) OR Resonance Elastography, Magnetic))) OR (((((((Sonoelastography) OR Sonoelastographies) OR Acoustic Radiation Force Impulse Imaging) OR ARFI Imaging) OR ARFI Imagings) OR Imaging, ARFI) OR Imagings, ARFI)) AND ((((Fibrosis, Liver) OR Fibroses, Liver) OR Liver Fibroses) OR Liver Fibrosis)
